# Effect of green coffee on miR-133a, miR-155 and inflammatory biomarkers in obese individuals

**DOI:** 10.1186/s13098-024-01478-7

**Published:** 2024-10-28

**Authors:** Naglaa F. Khedr, Enas S. Zahran, Abla M. Ebeid, Samuel T. Melek, Rehab H. Werida

**Affiliations:** 1 Biochemistry Department, Faculty of Pharmacy, Medical Complex, Tanta University, Al-Baher St, Tanta, 31527 Egypt; 2 Internal Medicine Department, Faculty of Medicine, Menoufia University, Shebeen Elkom, Egypt; 3 Clinical Pharmacy Department, Faculty of Pharmacy, AL-Delta University, Gamasa, Egypt; 4 Department of Parasitology and Blood Research at National Organization for Drug Control and Research (NODCAR), 12654, Cairo, Egypt; 5 Clinical Pharmacy Department, Faculty of Pharmacy, Damanhour University, Damanhour, Egypt

**Keywords:** Adiponectin, Green coffee, Metabolic syndrome, miR-133a, miR-155, Resistin

## Abstract

**Objectives:**

Metabolic syndrome is a cluster of conditions that increases the risk of atherosclerotic cardiovascular diseases. The current study was a randomized, double blind, placebo-controlled study that aimed to determine the impact of green coffee (GC) in obese patients with metabolic syndrome through analysis of miRNA-155, miRNA-133a and the inflammatory biomarkers such as resistin, TNF-α, total sialic acid, homocysteine, high sensitivity C-reactive protein (hs-CRP), and the anti-inflammatory cytokine, adiponectin.

**Methods:**

One hundred-sixty obese patients were randomly supplemented either with GC capsules (800 mg) or placebo daily for six months. Both groups were advised to take a balanced diet. Blood samples were collected at baseline and after six months of supplementation.

**Results:**

GC supplementation for 6 months reduced BMI (p = 0.002), waist circumference (p = 0.038), blood glucose (p = 0.002), HbA1c% (p = 0.000), Insulin (p = 0.000), systolic blood pressure (p = 0.005), diastolic BP (p = 0.001) compared with placebo. GC significantly decreased total cholesterol (TC, p = 0.000), LDL-C (p = 0.001), triglycerides (TG, p = 0.002) and increased HDL-C (p = 0.008) compared with placebo group. In addition, GC significantly (p ≤ 0.005) reduced total sialic acid, homocysteine, resistin, TNF-α, hs-CRP and the oxidative stress marker malondialdehyde (MDA), but increased serum adiponectin (p = 0.000) compared to placebo group. There was a significant reduction in the gene expression of miR-133a (p = 0.000) in GC group as compared with baseline levels and with the control placebo group (p = 0.001) after 6 months.

**Conclusion:**

GC administration modulated metabolic syndrome by decreasing BMI, high BP, blood glucose, dyslipidemia, miRNA-133a and inflammatory biomarkers that constitute risk factors for cardiovascular diseases.

*ClinicalTrials.gov registration No.* is NCT05688917.

**Graphical Abstract:**

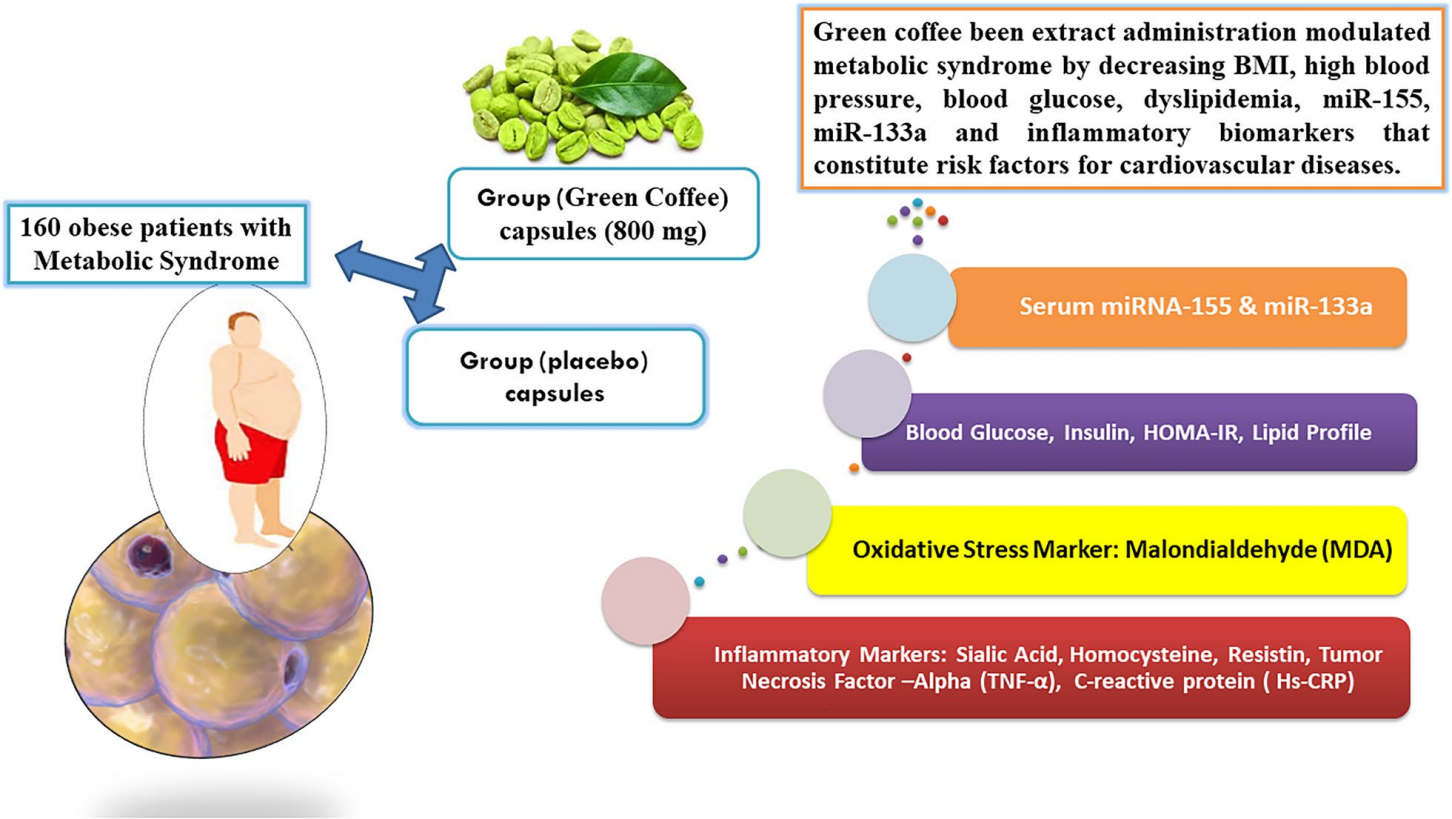

## Introduction

The metabolic syndrome (MetS) is associated with major risk factors for cardiovascular diseases (CVD) including diabetes, abdominal obesity, high cholesterol, and high blood pressure. The incidence of adults with MetS was about one third of USA adults [1]**.** MetS is usually characterized by secretion of inflammatory adipokines from adipose tissue that are mainly produced by infiltrating macrophages such as leptin, interleukin (IL-6), tumor necrosis factor-α (TNF-α), monocyte chemoattractant protein-1 (MCP-1), and resistin [2].

Obesity may cause systemic oxidative stress in accumulating fat cells. When reactive oxygen species (ROS) exceed antioxidant levels, it causes dysregulation of adipokines such as leptin and adiponectin that are released by adipose tissue. Excess adiposity leads to increased pro-inflammatory leptin production and decreased anti-inflammatory adiponectin levels [3]. Moreover, total adiposity and truncal subcutaneous fat accumulation are associated with increased risk of atherosclerosis at adult ages. However, accumulation of abdominal fat is associated with insulin resistance [4].

While inflammation is a necessary response to restore balance after various stressors, excessive or prolonged inflammation can be detrimental. Chronic low-grade inflammation is common in overweight and obese individuals while fat and liver tissues often show increased activation of certain kinases, like c-Jun N-terminal kinase and inhibitor of κB kinase, which trigger the production of inflammatory cytokines. These kinases influence downstream transcriptional processes through nuclear factor κB, interferon regulatory factor, and activator protein-1, leading to the up-regulation of genes that produce inflammatory mediators. This impairs metabolic pathways and exacerbates receptor activation. Additionally, the innate immune system’s Toll-like receptors and inflammasomes contribute to this chronic pro-inflammatory state and diminish insulin sensitivity [2, 5].

MicroRNAs play an important role in the regulation of inflammation and insulin resistance, two interrelated processes that lead to the development of a variety of metabolic illnesses such as obesity, type 2 diabetes, and CVD [6]. MiRNAs regulate both pro- and anti-inflammatory pathways. For example, miR-155 is essential modulators of inflammation that regulate immunological responses. Obesity was associated with increased expression of miR-155, which is activated by Nuclear Factor-kappa/Tumor Necrosis Factor (BNF-κB/TNF-alpha). MiR-155 mediates the chronic inflammatory state associated with obesity by promoting the expression of a wide range of genes and chemokines [7, 8].

Obesity-associated miRNAs have a direct impact on cardiovascular health because they affect cardiac remodeling, endothelial function, and vascular smooth muscle cell proliferation. Several microRNAs have been identified as important participants in these processes [8, 9]. For example, miR-133a is linked to unfavorable cardiac remodeling, such as hypertrophy and fibrosis [7]. MiR-133a-1, miR-133a-2, and miR-133b can inhibit insulin-stimulated glucose uptake in skeletal muscle and adipocytes [10]. Moreover, miR-133a regulates insulin-like growth factor 1 (IGF-1) in the heart and regulates the etiology of insulin resistance; it was found that IGF-1 depletion reduces the phosphorylation of GATA binding protein 4, glucose transporter-4 (GLUT4), and protein kinase B (AKT) [11].

The growing risk of obesity to global health has encouraged scientists to find natural compounds that help to decrease body weight and delay the development of CVD and type II diabetes. Green coffee bean extract (GC) is a supplement extracted from raw coffee beans prior to fermentation and roasting. It has antioxidant properties and reduces belly fat, body weight, incidence of cancer, diabetes and liver disease [12–14]. The bioactive compounds present in green coffee, not only chlorogenic acids and their derivatives, but also caffeine, theophylline, and theobromine, cafestol, kahweol, tocopherols and trigonelline [13, 14]. However, the antioxidant activity of coffee beans mainly depends on the phenolic compound chlorogenic acid (CGA). Previous studies reported that CGA improves insulin sensitivity, increases glucose uptake in skeletal muscle and modulates several metabolic pathways [14, 15].

GC extract rich in chlorogenic acid inhibited the inflammatory mediators and metastasis of colorectal cancer cell lines (SW480) via NF-κB inactivation [16]. In animal studies, GC been extract improved lipid profile, glucose homeostasis and insulin resistance. Also, it increased serum levels of adiponectin and the expression of GLUT4 expression in adipose tissue thus improving insulin resistance (IR) [17]**.** Additionally, the green coffee potential bioactivity maintain physiological homeostasis, enhance physiological balance in MetS conditions and display cardio-protective through regulating lipid and glucose metabolism, IR, inflammation, modulation of adenosine monophosphate-activated protein kinase (AMPK) and Peroxisome Proliferator-Activated Receptor α (PPARα) gene expression in MetS rat model and mice [18–20].

A recent study utilizing a metabolic approach found that treatment with GC bean extract, either alone or in combination with green tea extract, led to reductions in glucose, lactic acid, and urea levels, while increasing serum levels of oleamide, stearic acid, stearamide, and glycerol monostearate in obese rats fed a high-fat diet. These metabolites could serve as key biomarkers for investigating how GC ingestion may contribute to the treatment or prevention of obesity and its associated conditions. [3].

A randomized, double-blind clinical study showed significant changes in body fat, improved lipid profile in healthy overweight subjects with no adverse effects compared with placebo group [21]. Additionally, a randomized, double-blind, placebo-controlled trial with T2DM and overweight/obese patients showed beneficial effects of GC been extract on blood pressure, lipid profile, hs-CRP, and HDL-C levels over a 10 week period of supplementation [22].

Recent study also showed that 500 mg twice daily of a new, patented water-soluble green coffee bean extract (GCB70^®^), enhanced in 70% total chlorogenic acid and less than 1% caffeine, was given to 105 volunteers for 12 weeks. Body weight, BMI, waist circumference, lipid profile, plasma leptin, glycosylated hemoglobin (HbA1c %), and thyroid-stimulating hormone all significantly decreased by GCB70® treatment [23].

Even so, discrepant data are present on the impact of GC on obesity related inflammation. For this reason, the present study aimed to investigate the underlying molecular mechanisms of GC been extract on miRNA-133a and miRNA-155 that modulate insulin resistance, inflammation and combat CVD diseases. Moreover we explore the role of GC on the inflammatory biomarkers in obese patients with MetS as resistin, TNF-α, total sialic acid, homocysteine, high sensitivity C-reactive protein (hs-CRP) and the anti-inflammatory cytokine, adiponectin.

## Subjects and methods

### Subjects and study design

This is a prospective, randomized, double blind, placebo-controlled study, conducted between September 2020 and September 2022. Participants were collected from out-patient clinics at Menoufia University hospital, Egypt. Patients were enrolled if they were aged 20–60 year and meet the new International Diabetes Federation (IDF) definition of metabolic syndrome [24] including: waist circumference > 102 cm in male; 88 cm in female, triglycerides ≥ 150 mg/dL, HDL-C < 40 mg/dL in males & < 50 mg/dL in females, systolic BP ≥ 130 or diastolic BP ≥ 85 mm Hg and fasting blood sugar > 100 mg/dL.

### Exclusion criteria

The exclusion criteria were liver, kidney, coronary artery disease, type I diabetes, autoimmune diseases, cancer, infection, smoking, recent major surgery, routine coffee consumption, pregnancy or breast-feeding, use of anti-inflammatory drugs, corticosteroids, hormone replacement therapy, weight-loss and anti-hypercholesterolemic medications three months or less prior to enrolment.

**Fig. 1:**
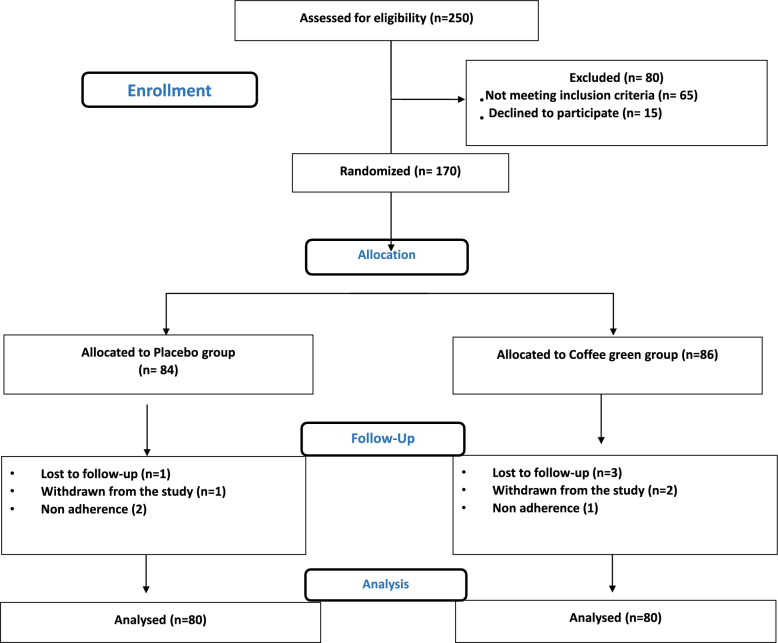
CONSORT Flow Diagram of the progress through the randomized study

Based on the exclusion criteria, 250 subjects were recruited from the endocrinology out-patient unit (Menoufia University Hospital, Egypt) and were considered eligible for the study **(**Fig. 1**)** between September 2020 to September 2022. All participants had an informed consent before entering the study and were subjected to a full clinical examination including anthropometric measurements and full medical history were obtained. Clinical and biochemical assessments were done at baseline and after six months of treatment. Body-mass index (BMI) = weight (kg)/height (m^2^) and waist circumference (WC) were recorded. Blood pressure was measured twice, after keeping participants in a sitting position for 15 min. The mean value of two consecutive measurements with 5 min intervals was used for study purposes. Only 160 participants maintained on the supplements or placebo and completed the study.

Participants included in the study were maintained on balanced healthy diet and supplemented either with Green Coffee Bean (*Coffea Arabica)* extract capsules (800 mg/capsule, standardized to contain 50% chlorogenic acid equivalent to 400 mg, Puritan`s Pride^®^, NY, USA) or placebo capsule once daily 30 min before lunch for six months. Bottles of GC supplements containing twenty-one capsules for 21 days were given to subjects on the first day of recruitment and the other bottles were given every 3 week follow up visit. Also, participants were followed by phone calls every week to ensure that they complied with study protocol and for any other inquiry about the supplementation. The GC dose was chosen based on previous research [21] that supports the anti-obesity benefits of 500 mg of green coffee beans extract in overweight persons, as well as its efficacy and safety for intake as a functional food component. Furthermore, consuming 800 mg/day of GC been extract may ameliorate dyslipidemia in obese people [25]**.**

### Characters of the balanced diet

According to WHO criteria [26], participants were advised to take a balanced diet not exceed 2000 kcal/day to maintain their requirements from macronutrients, vitamins and minerals. The diet should include at least 400 g fruit, vegetables, legumes, nuts and whole grains. Less than 10% of total energy intake from free sugars (50 g) and less than 30% of total energy intake from fats (unsaturated fats are preferable) and less than 5 g of iodized salt per day should be included.

### Biochemical investigations

Blood samples were drawn after overnight fasting (12 h). Blood samples were divided into three tubes; a plain serum tubes for biochemical analysis and EDTAed tubes for HbA1c %, and Tempus^™^ Blood tubes for RNA stabilization that stored at− 80 °C until RNA extraction. The serum was separated after centrifugation at 800 × g for 10 min and kept frozen at− 80 °C until biochemical analysis. Blood glucose [27], malondialdehyde (MDA) [28], triglycerides (TGs) [29], total cholesterol (TC) [30], and high-density lipoprotein cholesterol (HDL-C) [31] were determined by enzymatic colorimetric assay using kits purchased from Biodiagnostics Company (Giza, Egypt). Low-density lipoprotein- cholesterol (LDL-C) was calculated according to Friedewald`s formula [32]. HbA1c% was determined using EDTAed blood by ion exchange method using kits obtained from Stanbio Laboratory Company (USA). Serum insulin was determined by ELISA (Insulin ELISA kit human, KAQ1251, Invitrogen Corporation, Camarillo, CA, USA). Homeostasis Model Assessment of Insulin Resistance (HOMA-IR) was calculated using the formula: HOMA-IR = [glucose (mg/dL) × insulin (IU/L)/405], using fasting values [33]. Serum total sialic acid was determined photometrically at 340 nm [34] using SIALICQ quantitation kit (sigma Aldrich (St. Louis, MO, USA)**.** Serum homocysteine was assayed fluorometrically using Kit (ab228559, Cambridge, MA, USA). Serum high-sensitivity CRP (hsCRP) was determined by immunonephelometry (Dade Behring, BNII, Marburg, Germany). Serum adiponectin, resistin and TNF-α concentrations were measured using Human Adiponectin ELISA (RayBiotech Company, USA), Human Resistin and TNF-α ELISA Kits (Invitrogen, ThermoFisher scientific, Austria**),** respectively as described by the manufacturer` protocol**.**

### Reverse transcription quantitative PCR (RT-qPCR) of miR-133a and miR-155

Total RNA was extracted with a QIAZOL reagent and the miRNeasy Serum/Plasma isolation kit (Qiagen, catalog number 217184, USA). RNA content and purity were determined using spectrophotometry with NanoDrop® (Analytikjena Scandrop 200 Germany). The miScripT II RT kit (Qiagen, catalogue no. 21860, USA) was used to convert total RNA to cDNA, as directed by the manufacturer (Qiagen, CA, USA). miRNAs were measured using RT-qPCR with a 7900HT Fast Real-Time PCR System (Applied Biosystems) and the miScript SYBR Green Quantitative PCR kit (Qiagen, catalogue no. 218073 Foster City, CA, USA). The qPCR procedure consisted of 5 min at 95 °C, followed by 40 cycles (94 °C for 10 s, 55°C for 20 s, 72°C for 10 s, and 80°C for 35 s. All cDNA samples were taken in duplicate. Gene sequences were acquired from the miRBase database (http://www.mirbase.org/). The housekeeping gene, RNU43, was utilized to standardize each miRNA's expression levels. The sequences of the primers [35, 36] that were produced by Biosearch Technologies Co. (USA) are as follows: RNU43-F: 5′-GTGAACTTATTGACGGGCG-3`, RNU43-RT: 5′-GTTGGCTCTGGTGCAGGGTCCGAGGTATTCGCACCAGAGCCAACAATCAG-3` and TATGCTTGTTCTCGTCTCTGTGTC-3′; miR-155-3p-F 5′-GTTTGGCTCCTACATATTAGCA-3′, miR-155-3p-R: 5`- GTTGGCTCTGGTGCAGGGTCCGAGGTATTCGCACCAGAGCCAACTGTTAA-3`, miR-133-3p-F: 5′-ACACTCCAGCTGGGTTTGGTCCCCTTCAAC-3′ and miR-133-3p-R: 5′-CTCAACTGGTGTCGTGGAGTCGGCAATTCAGTTGAGCAGCTGGT-3′. The fold change in miRNA expression was estimated using the comparative CT method as fold change = 2—ΔΔCT, where ΔΔCT = ΔCT sample ˗ ΔCT control [37].

### Clinical outcomes

We identified the onset of any cardiovascular event or vascular outcomes, or mortality during follow-up to assess the effectiveness of the treatment. We also assessed any incident pneumonia or urinary tract infections.

### Adverse effects

Any stomach upset, anxiety, headaches, rapid heartbeats, jitteriness, that occurred after the initiation of treatment due to caffeine component of GC. The data was recorded and calculated as (%) using the following formula: number of patients with side effect/total patient number × 100.

### Data collection sheet

For this study, a systematic data collection sheet was created. The measurements included inquiries about the patient's sociodemographic information (including age, sex, height, weight, and BMI), medical history and concomitant conditions, lifestyle choices, lab tests, and adverse effects.

### Statistical analysis

The collected data were analyzed using software statistical computer package SPSS version 26.0 (SPSS Inc, Chicago, IL, USA) [38]. The total sample size of 160 patients was calculated using G*Power software version 3.1.0 (Institute fur Experimentelle Psychologie, Heinrich Heine Universitat, Dusseldorf, Germany) which had a power of 96%. Qualitative data were expressed as frequency and percentage. Data were tested for normality using Kolmogrov–Smirnov and Shapiro–Wilk test. The Chi-square test was used to compare categorical data between the two studied groups. Student t-test used for normally distributed quantitative variables and numerical data were expressed as mean and standard deviation. Non-parametric data were expressed as median (Min–Max) and were analyzed using Wilcoxon signed-rank test to compare before and after the intervention and Mann–Whitney U test to compare between both studied groups. Spearman’s correlation was used for analysis of the bivariate relationship. Receiver operating characteristic (ROC) curve used to compare performance between two tests. P-values < 0.05 were considered significant.

## Results

### Demographic characteristics of included patients

In Table 1 shows the demographic characteristics of included patients in the control placebo group and GC group that showed non-significant differences at baseline before intervention. Patients in the control group were aged 45.51 ± 8.51 *vs.* 44.28 ± 9.56. Control group included 39 (48.8%) males and 41 (53.1%) females *vs.* 36 (45%) males and 44 (55%) females in GC group. There were non-significant differences of the duration of diabetes between the two groups. The associated diseases and medical history of control group *versus* GC supplemented group were as follows; osteoarthritis (20% *vs.* 23.8 %), hypertension (37.5% *vs*. 42.5%) & dyslipidemia (40% vs. 35.5%). Moreover, twenty-three patients of control group *vs.* twenty-five of GC group were treated with oral hypoglycemic agents (metformin, sulfonylurea, and α-Glucosidase inhibitors). Also, 35 patients of control group *vs*. 39 patients of GC group were treated with antihypertensive medications (angiotensin converting enzyme inhibitors, angiotensin receptor blockers and B-blockers) with non-significant differences between both groups.

**Table 1:** Demographic Characteristics of patients

Baseline variables	Control (n = 80)	Coffee green (n = 80)	P value
Age (years)	45.51 ± 8.51	44.28 ± 9.56	0.388
Sex			
Male	39 (48.8)	36 (45)	0.635
Female)	41 (53.1)	44 (55)	
Height (cm)	171.28 ± 7.87	172.28 ± 8.47	0.440
Associated diseases			
Mild Osteoarthritis	16 (20)	19 (23.8)	0.566
HTN	30 (37.5)	34 (42.5)	0.519
Dyslipidemia	32 (40.0)	28 (35.5)	0.514
Smoker	20 (25.0)	22 (27.5)	0.719
Oral hypoglycemic drugs (n) (%)			
Metformin	30 (37.5)	32 (40)	0.746
Sulfonylureas	25 ( 31.3)	21(26.3)	0.485
α-Glucosidase inhibitors	25(31.3)	27 (33.8)	0.736
Anti-hypertensive drugs			
ACE inhibitors	14 (17.5)	15 (18.8)	0.837
ARB	14 (17.5)	16 (20.00)	0.685
B-blockers	7 (8.8)	8 (10.0)	0.786
Other medications			
Librax ®	7 (8.8)	5 (6.3)	0.548
Thiotacid ®	11 (13.8)	9 (11.3)	0.633
Duration of diabetes			
< 5 years	69 (86.3)	71 (88.8)	0.633
5–10 years	11 (13.8)	9 (11.3)	

Data are presented as Mean ± SD, number (%). Total number of participants is n = 160

*HTN* hypertension, *ACE *inhibitors: Angiotensin converting enzyme inhibitors, *ARBs* Angiotensin receptor blockers. Data analyzed using Independent T-test or Chi-square as appropriate

p < 0.05 was set as significant

### Effect of green coffee supplementation on body weight, BMI, WC, blood pressure, lipid profile and miRNAs

In Table 2 shows the effect of GC supplementation with balanced diet on obesity indicators, blood pressure and lipid profile after 6 months**.** Body weight, BMI, WC, systolic BP, diastolic BP, TC, LDL-C and TG were decreased significantly in both groups compared to baseline. Compared to baseline, green coffee group showed a significant decrease in body weight (84.36 ± 10.09 *vs.* 93.60 ± 11.00, p = 0.000), BMI (28.49 ± 3.48 *vs*. 31.59 ± 3.61, p = 0.000), as well as WC (97.41 ± 13.85 *vs.* 104.59 ± 13.32, p = 0.000) after 6 months of treatment **(**Table 2**)**.

**Table 2:** Effect of green coffee supplementation on body weight, BMI, WC, blood pressure, lipid profile and miRNAs

	Control group (n = 80)			Coffee green group (n = 80)				
	Baseline	After 6 months	*P*^*a*^* value*	Baseline	After 6 months	*P*^*a*^	P^b^ baseline	P^c^ after
Body weight (Kg)	90.81 ± 15.45	89.29 ± 14.43	*0.000*	93.60 ± 11.00	84.36 ± 10.09	*0.000*	*0.191*	*0.013*
BMI (kg/m^2^)	30.93 ± 4.57	30.42 ± 4.27	*0.000*	31.59 ± 3.61	28.49 ± 3.48	*0.000*	*0.315*	*0.002*
wC (cm)	105.92 ± 11.30	101.53 ± 10.79	*0.000*	104.59 ± 13.32	97.41 ± 13.85	*0.000*	*0.497*	*0.038*
Systolic BP (mmHg)	138.25 ± 12.47	129.50 ± 6.36	*0.000*	137.64 ± 13.94	126.20 ± 8.04	*0.000*	*0.770*	*0.005*
Diastolic BP (mmHg)	87.44 ± 3.38	83.93 ± 3.62	*0.000*	86.65 ± 2.54	81.66 ± 4.45	*0.000*	*0.098*	*0.001*
TC (mg/dL)	211.89 ± 24.80	199.04 ± 31.89	*0.000*	215.04 ± 32.95	182.24 ± 23.9	*0.000*	*0.495*	*0.000*
LDL-C (mg/dL)	137.99 ± 24.99	128.33 ± 31.08	*0.000*	141.12 ± 33.05	114.42 ± 22.58	*0.000*	*0.499*	*0.001*
HDL-C (mg/dL)	42.11 ± 2.60	41.54 ± 4.56	*0.229*	41.54 ± 2.41	43.10 ± 2.44	*0.000*	*0.149*	*0.008*
TG (mg/dL)	158.93 ± 9.07	145.90 ± 24.30	*0.000*	161.89 ± 14.03	123.59 ± 22.44	*0.000*	*0.115*	*0.002*
miR-133a (RCN)	0.91 (0.14–2.01)	0.90 (0.20–1.50)	*0.000*	0.94 (0.34–2.59)	0.67 (0.27–1.40)	*0.000*	*0.384*	*0.001*
miR-155 (RCN)	1.24 (0.20–3.78)	1.20 (0.22–3.40)	*0.090*	1.14 (0.33–3.65)	1.10 (0.30–3.40)	*0.000*	*0.375*	*0.218*

Values shown are means ± S.D or median (Min–Max)

P < 0.05 was set as significant

P values are in italics

*BMI* body mass index = weight (kg)/height (m^2^), *WC* waist circumference (cm), *TG* Triglycerides, *TC* total cholesterol, LDL-C: low density lipoprotein-cholesterol, HDL-C: high density lipoprotein-cholesterol. RCN: relative copy Number, Green coffee bean extract (800 mg) was given daily for 6 months. P^a^ Paired T test or Wilcoxon Signed Ranks Test, P^b^ Independent T test or Mann–Whitney U baseline, P^c^ Independent T test or Mann–Whitney U after intervention

In Table 2 also shows that GC supplementation along with balanced diet modulated blood pressure; sBP was decreased (126.20 ± 8.04 *vs.* 137.64 ± 13.94; p = 0.000) as well as dBP (81.66 ± 4.45 *vs.* 86.65 ± 2.54; p = 0.000) compared to baseline assessment**.** Patients included in the present study showed dyslipidemia which was modulated after GC supplementation for 6 months. As shown in Table 2, there was significant decrease in TC (182.24 ± 23.9 *vs.* 215.04 ± 32.95; p = 0.000), LDL-C (114.42 ± 22.58 *vs.* 141.12 ± 33.05; p = 0.000), triglycerides (123.59 ± 22.44 *vs.* 161.89 ± 14.03; p = 0.000) and increased HDL-C (43.10 ± 2.44 *vs.* 41.54 ± 2.41; p = 0.000) after green coffee supplementation compared with baseline assessment.

Table 2 shows that control-placebo group maintained on balanced diet for six months exhibited decreased BMI, WC, blood pressure and corrected dyslipidemia when compared to baseline assessment. BMI was decreased (30.42 ± 4.27 *vs.* 30.93 ± 4.57; p = 0.000), sBP (129.50 ± 6.36 *vs.* 138.25 ± 12.47, p = 0.000), dBP (83.93 ± 3.62 *vs.* 87.44 ± 3.38, p = 0.000), TC (199.04 ± 31.89 *vs.* 211.89 ± 24.80, p = 0.000), LDL-C (128.33 ± 31.08 *vs.* 137.99 ± 24.99, p = 0.000) and TG (145.90 ± 24.30 *vs.* 158.93 ± 9.07, p = 0.000) with non-significant changes in HDL-C compared to baseline assessment. Table 2 reveals significant differences between the control group and green coffee group after 6 months of intervention (p < 0.001).

### Effect of green coffee supplementation on fasting blood glucose, serum insulin and HOMA-IR

**Fig. 2:**
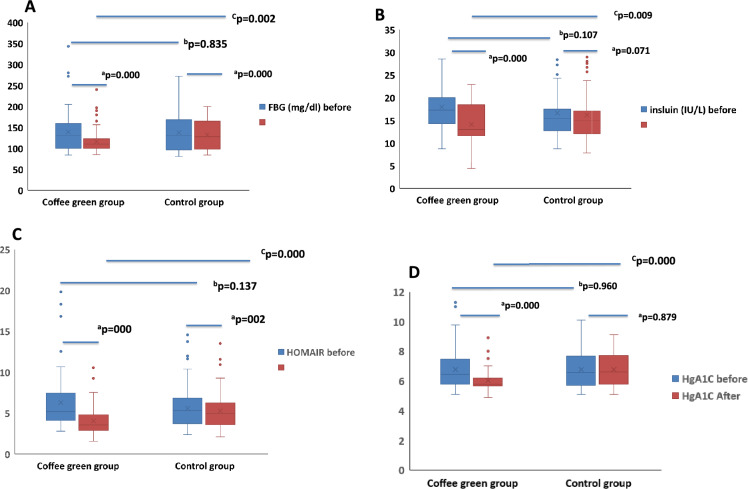
Box-and-Whisker plots of Fasting blood glucose (**A**), Serum Insulin (**B**), HOMA-IR (**C**) and HbA1c % (**D**) were significantly improved by GCBE supplementation. Box-and-Whisker plots show the upper and lower quartiles and range (box), median value (horizontal line inside the box), and full range distribution (Whisker line). The paired t-test was used to evaluate the statistical significance of GCBE treatment. HOMA-IR = [glucose (nmol/L) × insulin (μU/mL)/22.5], using fasting values

In Box-and-Whisker plots (Fig. 2**)** show the upper and lower quartiles and range (box), median values (horizontal line inside the box), and full range distribution of FBG, fasting serum insulin, HOMA-IR and HbA1c%. Figure 2 shows that GC supplementation for six months improved FBG, HbA1c %, serum insulin and insulin resistance (HOMA-IR) in GC group; FBG was decreased (116.34 ± 28.00 mg/dL *vs.* 139.16 ±  47.07; p = 0.000), HbA1c% (6.05 ± 0.73 *vs.* 6.78 ± 1.30; p = 0.000), insulin (14.09 ± 4.76 IU/L *vs.* 17.97 ± 4.99; p = 0.000) and HOMA-IR (4.04 ± 1.74 *vs.* 6.29 ± 3.28; p = 0.000) compared to baseline levels in GC group. Green coffee supplementation also showed significant decrease in FBG (116.34 ± 28.00 mg/dL *vs.*132.49 ± 36.07, p = 0.002), serum insulin (14.09 ± 4.76 IU/L *vs.*16.14 ± 5.1, p = 0.009), HbA1c% (6.05 ± 0.73 *vs.* 6.76 ± 1.08, p = 0.000) and HOMA-IR (4.04 ± 1.74 *vs.*5.27 ± 2.29, p = 0.000) compared to control placebo group after 6 months. However, the control group, which followed a balanced diet, showed non-significant changes in serum insulin and HbA1c% but showed significant lower FBG levels (132.49 ± 36.07 *vs.* 137.66 ± 43.79, p = 0.000) and HOMA-IR (5.27 ± 2.29 *vs.* 5.60 ± 2.50, p = 0.002) after 6 months of balanced diet.

### Effect of green coffee supplementation on gene expression of miR-133a and miR-155

**Fig. 3:**
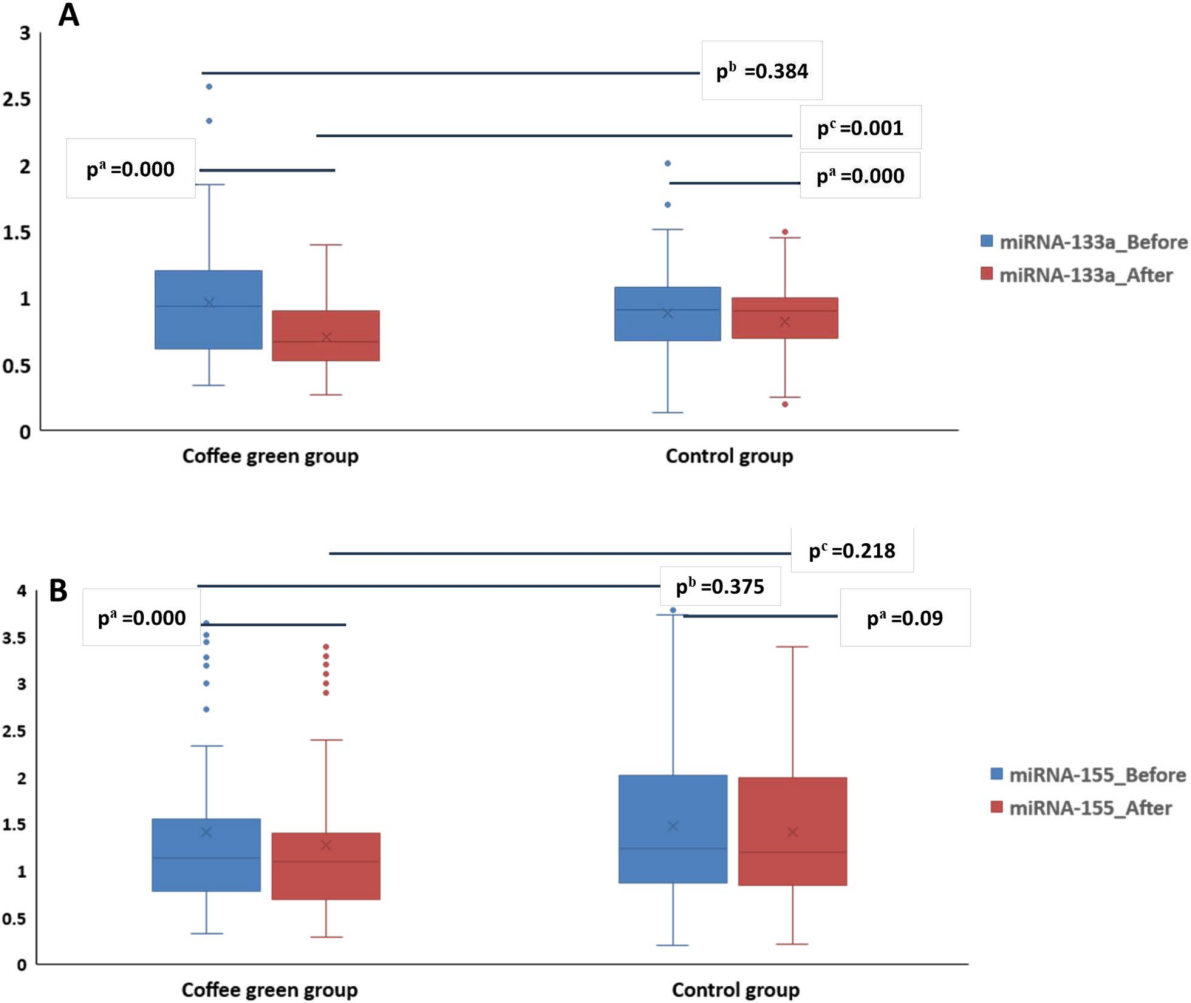
Box-and-Whisker plots of miRNA-133a (**A**), miRNA-155 (**B**) gene expression as fold change, in studied groups

Figure 3A shows that gene expression of miR-133a was significantly reduced by 0.71 fold (p^a^ = 0.000) in participants of GC group after 6 months treatment [0.67 (0.27–1.40)] compared with baseline levels [0.94 (0.34–2.59)]. In addition, GC group after 6 months treatment [0.67 (0.27–1.40)] showed a significant decrease in mRNA-133a gene expression by 0.74 fold (p^c^ = 0.001) compared with placebo group after 6 months of intervention [0.90 (0.20–1.50)] **(**Fig. 3A**).**

Figure 3B shows that gene expression of miR-155 as fold change. Treatment with GC for 6 months [1.10 (0.30–3.40)] significantly reduced miR-155 expression by 0.96 fold (P^a^ < 0.000) compared to baseline levels [1.14 (0.33–3.65)]. However, non-significant change was observed between GC group and placebo group at baseline (p^b^ = 0.375) and after the intervention (p^c^ = 0.218) of mRNA expression levels.

### Effect of green coffee supplementation on inflammatory and oxidative stress biomarkers

As expressed in Table 3, the effect of GC and balanced diet on the inflammatory and oxidative stress biomarkers revealed that total sialic acid, homocysteine, resistin, TNF-α, hs-CRP and MDA were decreased significantly when compared to baseline assessment. On the other hand, adiponectin was increased after supplementation as compared to baseline. GC decreased serum total sialic acid (3.36 ± 1.69 *vs*. 5.61 ± 2.49, p = 0.000), homocysteine (10.73 ± 3.14 *vs.* 14.11 ± 3.63, p = 0.000), resistin (17.36 ± 1.64 *vs.* 27.43 ± 3.80, p = 0.000), TNF-α (6.56 ± 1.95 *vs.* 11.95 ± 3.16; p = 0.000), hs-CRP by 36.19% (17.08 ± 1.12 *vs.* 25.15 ± 4.89, p = 0.000) and MDA (1.34 ± 0.65 *vs.* 4.10 ± 0.36, p = 0.000), whereas serum adiponectin was increased after GC supplementation (6.22 ± 1.40 *vs.* 2.58 ± 0.41; p = 0.000) compared to baseline assessment.

**Table 3:** Effect of green coffee supplementation on inflammatory and oxidative stress biomarkers

	Control Group (n = 80)			Coffee Green Group (n = 80)				
	Baseline	After 6 months	*P*^*a*^* value*	Baseline	After 6 months	*P*^*a*^* value*	P^b^	P^c^
Total sialic acid (mmol/L)	6.21 ± 2.22	7.20 ± 2.37	*0.000*	5.61 ± 2.49	3.36 ± 1.69	*0.000*	*0.108*	*0.000*
Homocysteine (µmol/L)	13.59 ± 1.40	12.11 ± 2.98	*0.000*	14.11 ± 3.63	10.73 ± 3.14	*0.000*	*0.235*	*0.005*
Resistin (ng/mL)	28.17 ± 1.99	20.23 ± 4.25	*0.000*	27.43 ± 3.80	17.36 ± 1.64	*0.000*	*0.128*	*0.000*
Adiponectin (µg/mL)	2.53 ± 0.51	5.10 ± 1.99	*0.000*	2.58 ± 0.41	6.22 ± 1.40	*0.000*	*0.493*	*0.000*
TNF-α (pg/mL)	11.67 ± 2.82	10.70 ± 30.06	*0.000*	11.95 ± 3.16	6.56 ± 1.95	*0.000*	*0.555*	*0.000*
Hs-CRP (mg/L)	25.97 ± 3.71	18.39 ± 3.66	*0.000*	25.15 ± 4.89	17.08 ± 1.12	*0.000*	*0.235*	*0.003*
MDA (µmol/L)	4.09 ± 0.33	1.85 ± 1.08	*0.000*	4.10 ± 0.36	1.34 ± 0.65	*0.000*	*0.879*	*0.000*

Values shown are means ± SD

p < 0.05 was set as significant. Data analyzed using Independent T-test

P values are in italics

*P*^*a*^ Paired T test, *P*^*b*^ Independent T test baseline, *P*^*c*^ Independent T test after intervention. *TNF-α* Tumor necrosis factor-alpha, *MDA* malondialdehyde, *Hs-CRP* High sensitivity C-reactive protein. *GCBE* Green coffee bean extract (800 mg) daily for 6 months

Table 3 also shows the effect of balanced diet in placebo group for 6 months on the inflammatory and oxidative stress biomarkers. There was significant decrease in serum homocysteine (12.11 ± 2.98 *vs.* 13.59 ± 1.40, p = 0.000), resistin (20.23 ± 4.25 *vs.* 28.17 ± 1.99, p = 0.000), TNF-α (10.70 ± 30.06 *vs.* 11.67 ± 2.82, p = 0.000), hs-CRP (18.39 ± 3.66 *vs.* 25.97 ± 3.71, p = 0.000), MDA (1.85 ± 1.08 vs. 4.09 ± 0.33, p = 0.000) compared to baseline assessment. However, total sialic acid (7.20 ± 2.37 vs. 6.21 ± 2.22, p = 0.000) and adiponectin (5.10 ± 1.99 vs. 2.53 ± 0.51, p = 0.000) were increases as compared to baseline assessment. Moreover, significant differences (p < 0.002) were observed between the placebo group maintained on balanced diet only and coffee green supplemented groups after 6 months (Table 3).

### Area under ROC curve of measured parameters after green coffee supplementation

**Fig. 4:**
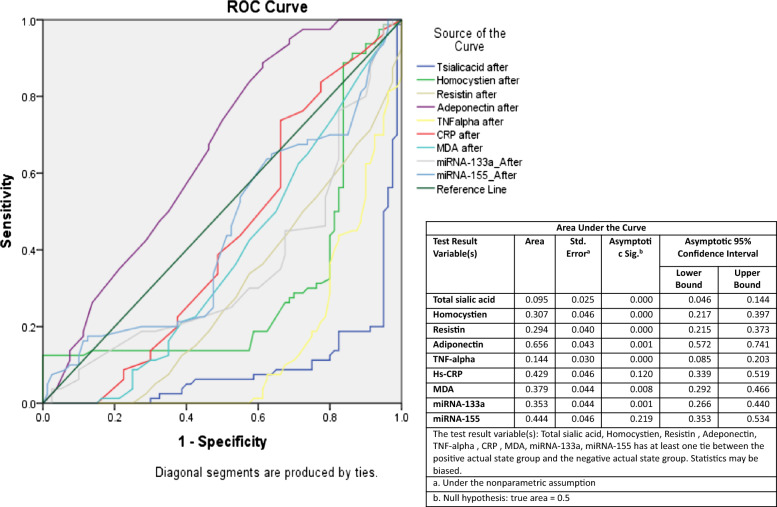
Area under ROC curve of measured parameters after green coffee treatment

Figure 4 shows the ROC-AUC of the measured biomarkers of GC group after 6 months treatment. Adiponectin was the most sensitive (AUC = 0.66, *p* = 0.001) followed by miR-155 (AUC = 0.44, p = 0.22), hs-CRP (AUC = 0.43, p = 0.12), MDA (AUC = 0.38, p = 0.008), miR-133a (AUC = 0.35, p = 0.001), homocysteine (AUC = 0.31, p = 0.000), resistin (AUC = 0.29, p = 0.000), TNF-α (AUC = 0.14, p = 0.000) and total sialic acid (AUC = 0.10, p = 0.000).

### Correlation study between the measured parameters

Table 4 shows the correlation between miR-133a & miR-155 expression, and glucose homeostasis biomarkers after treatment with green coffee for 6 months. MiR-133a was positively correlated with FBG (r = 0.18, p = 0.02) and HbA1C (r = 0.2, *p* = 0.01). In Addition, miR-155 was positively correlated with FBG (r = 0.3, p = 0.000), HbA1C (r = 0.28, p = 0.000) and HOMA-IR (r = 0.319, p = 0.000).

**Table 4:** Correlation study between miR-133a, miR-155 and glucose homeostasis biomarkers after treatment with green coffee for 6 months

	HbA1C %		insulin (IU/L)		HOMA-IR		miRNA-133a		miRNA-155	
	*r*	*p*	*r*	*p*	*r*	*p*	*r*	*p*	*r*	*p*
FBG(mg/dl)	0.904^**^	*0.000*	0.036	*0.651*	0.636^**^	*0.000*	0.180*	*0.023*	0.300^**^	*0.000*
HbA1_C_ %			0.038	*0.634*	0.589^**^	*0.000*	^*^	*0.011*	0.278^**^	*0.000*
Insulin					0.765^**^	*0.000*	-0.030	*0.707*	0.140	*0.077*
HOMA-IR							0.123	*0.121*	0.319^**^	*0.000*
miRNA-133a									0.158^*^	*0.045*

HOMA-IR = [glucose (mg/dL) × insulin (IU/L)/405], **. Correlation is significant at the 0.01 level (2-tailed). *. Correlation is significant at the 0.05 level (2-tailed)

P values are in italics

As shown in Table 5, total sialic acid was positively and significantly (p = 0.01) correlated with homocysteine, resistin, TNF-α, hs-CRP and miR-155. In addition, there was a positive correlation between resistin and homocysteine, TNF-α, hs-CRP and MDA. Also, adiponectin was negatively correlated (p < 0.05) with sialic acid, resistin, TNF-α, hs-CRP, MDA. MiR-133a was positively correlated TNF-α & miR-155. However, miR-155 positively and significantly (p = 0.004) correlated with sialic acid (Table 6).

**Table 5:** Correlation study between miR-133a & miR-155 expression, inflammatory and oxidative stress biomarkers after treatment with green coffee for 6 months

	Homocysteine		Resistin		Adiponectin		TNF- α		CRP		MDA		miRNA-133a		miRNA-155	
	*r*	*p*	*r*	*p*	*r*	*p*	*r*	*p*	*r*	*p*	*r*	*p*	*r*	*p*	*r*	*p*
T sialic acid	0.36^**^	*0.00*	0.31^**^	*0.00*	-0.18^*^	*0.02*	0.417^**^	*0.000*	0.22^**^	*0.006*	0.14	*0.08*	0.12	*0.14*	0.224^**^	*0.004*
Homocysteine			0.18^*^	*0.02*	-0.09	*0.24*	0.119	*0.134*	0.15	*0.05*	0.067	*0.4*	-0.11	*0.15*	0.002	*0.98*
Resistin					-0.77^**^	*0.00*	0.349^**^	*0.000*	0.46^**^	*0.00*	0.72^**^	*0.00*	-0.01	*0.89*	0.10	*0.20*
Adiponectin							-0.23^**^	*0.002*	-0.270^**^	*0.001*	-0.75^**^	*0.00*	-0.016	*0.83*	0.002	*0.98*
TNF- α									0.307^**^	*0.000*	0.19^*^	*0.02*	0.17^*^	*0.03*	0.11	*0.17*
Hs-CRP											0.35^**^	*0.00*	0.03	*0.65*	0.12	*0.14*
MDA													0.05	*0.48*	0.04	*0.62*
miRNA-133a															0.16^*^	*0.04*

*TNF-α* Tumor necrosis factor-alpha, *MDA* malondialdehyde, *hs-CRP* High sensitivity C-reactive protein. Data analyzed using Independent T-test, * p < 0.05, ** p < 0.001

P values are in italics

^**^Correlation (r) is significant (*p*) at the 0.01 level (2-tailed). *. Correlation (r) is significant (*p*) at the 0.05 level (2-tailed)

**Table 6:** Side effects in both groups

Side effects	Control group (n = 80)	Coffee green group (n = 80)	P value
Headache	3 (3.8%)	6 (7.5%)	*0.303*
Gastric upset	2 (2.5%)	7 (8.8%)	*0.086*
Anxiety, agitation	2 (2.5%)	3 (3.8%)	*0.650*
Irregular beats	1 (1.9%)	2 (2.5%)	*0.560*

The data was recorded and calculated as (%) using the following formula: number of patients with side effect/total patient number × 100. Data analyzed using Chi-square test

P values are in italics

### Adverse effects and outcomes

Throughout the study, there were no significant differences in side effects between the two groups (p > 0.05). Vital sign measurements revealed no significant changes from the start of the study to the end of treatment. These findings suggested that GC supplementation was well tolerated at a daily dose of 800 mg.

## Discussion

Obesity is linked to insulin resistance and metabolic syndrome and contributes to conditions such as hypertension, high serum cholesterol, low HDL cholesterol, and elevated blood sugar levels. It also independently increases the risk of cardiovascular diseases, type II diabetes, and certain cancers. Moreover, excess fat accumulation in adipose tissues prompts these tissues to release inflammatory substances, leading to a pro-inflammatory state and oxidative stress [3].

To date, no research has investigated the effects of green coffee (GC) supplementation on miRNA-133a, miRNA-155, and various inflammatory markers associated with obesity and metabolic syndrome. This study is the first to examine this potential, providing new insights into how GC supplementation might influence these biomarkers and aid in managing obesity-related health problems. To ensure that all participants receive adequate nutrition, both the placebo and green coffee groups were provided with a balanced diet to meet their needs for vitamins, minerals, and macronutrients.

In this study, patients who received 800 mg of GC daily for 6 months, alongside with a balanced diet, showed improvements in BMI, blood pressure, blood glucose levels, and HOMA-IR, and experienced a correction in dyslipidemia compared to those in the placebo group. Moreover, numerous studies in both humans and animals have highlighted the positive effects of green coffee on glucose and lipid metabolism, benefiting both healthy individuals and those with genetic metabolic disorders [39] which were in accordance with our findings. Moreover, there was heterogeneity between studies when considering the effect of GC on HOMA-IR status. However, Meng et al. [40] reported that a dose greater than 400 mg of GC significantly decreases HOMA-IR.

GC contains chlorogenic acid (CGA) as its primary phenolic compound, which is known for its antioxidant properties. CGA functions as a hypoglycemic agent through several mechanisms: (a) it enhances insulin sensitivity and action, similar to metformin; (b) it inhibits the activity of hepatic glucose-6-phosphatase; (c) it reduces intestinal glucose absorption; and (d) it stimulates glucose uptake in both insulin-sensitive and insulin-resistant adipocytes. Additionally, unlike thiazolidinediones or insulin, CGA does not lead to weight gain or other adverse side effects [41]**.**

The impact of GC on lipid profile is primarily driven by CGA. CGA lowers total cholesterol by inhibiting lipid and cholesterol absorption in the intestines, reducing their transfer, and limiting hepatic biosynthesis [20]. Additionally, CGA enhances the expression of the PPAR-α gene and increases levels of carnitine palmitoyltransferase-1, while decreasing the expression of lipogenic factors such as sterol regulatory element-binding proteins (SREBPs). These SREBPs are crucial in regulating genes involved in glucose and lipoprotein metabolism, as well as liver inflammation [42, 43]. Clinically, synthetic PPAR-α agonists are used to treat dyslipidemia by lowering triglyceride levels and raising serum HDL-C levels, similar to the effects observed with CGA [20].

The current study found that markers of inflammation—specifically total sialic acid, homocysteine, resistin, TNF-α, hs-CRP—and the oxidative stress biomarker MDA were elevated at baseline compared to standard reference values, whereas adiponectin levels were lower. Additionally, a positive correlation was observed among these biomarkers.

Obesity is associated with a chronic, low-grade inflammatory state, characterized by elevated levels of pro-inflammatory cytokines such as TNF-α, IL-6, and CRP [44]. Macrophages` infiltration to fat tissue leads to overproduction of pro-inflammatory chemokines. This results in aggravation of localized inflammation in adipose tissue and spreading of an overall systemic inflammation that is associated with the development of obesity-related comorbidities [45]**.**

Furthermore, obese patients show increased activity of liver c-jun N-terminal kinase, which triggers the expression of inflammatory cytokines. These cytokines then enhance the activation of transcription factors like activator protein-1, nuclear factor-κB (NF-κB), and interferon regulatory factors, leading to a reduction in insulin sensitivity [46].

In this study, administering 800 mg of GC along with a balanced diet for six months led to a decrease in total sialic acid levels compared to a placebo. This effect may be linked to improvements in lipid profile, glucose regulation, or insulin sensitivity. Additionally, the CGA component in GC helps reduce inflammation and oxidative damage, which in turn lowers the release of sialic acid.

Sialic acid (SA), also known as N-acetyl-Neuraminic acid, is released from glycoconjugates by neuraminidase and plays various physiological roles. Inflammatory and oxidative stress conditions stimulate hepatocytes to produce SA, which then acts as a signaling molecule. This signaling can trigger myocardial injury by activating the Rho/ROCK-JNK/ERK signaling pathway [45]. Increased levels of sialic acid (SA) are linked to dyslipidemia, insulin resistance, and immune responses. Additionally, desialylated LDL is more susceptible to oxidative modification and greater accumulation compared to native LDL, which plays a role in the development of atherosclerosis [46].

Hyperhomocysteinemia accelerates atherothrombosis by elevating oxidative stress and impairing vascular endothelial function. Furthermore, it has been reported that homocysteine induces insulin resistance in vitro by inhibiting insulin signaling, with this effect being mediated by oxidative stress [47]. Herein, the present study showed a significant decrease in homocysteine level in coffee green group. These results were consistent with Ochiai et al*.* [48], who found that ingestion of GC decreases blood homocysteine level and improve vascular endothelial function.

Resistin is a peptide hormone secreted from human monocytes and macrophages and is involved in insulin resistance by impairing glucose tolerance. Moreover, resistin is correlated to abdominal fat depots and inflammation mediated by macrophages [49]. In this study, supplementation with GC for 6 months led to a reduction in resistin levels. These findings align with the work of Hwang et al. [50] and Huang et al. [51], who reported that CGA has anti-inflammatory effects by down-regulating iNOS, IL-1β, TNF-α, IL-6, and the chemokine CXCL1. Additionally, our results demonstrated a positive correlation between resistin and each of TNF-α, hs-CRP, total sialic acid, and homocysteine. This is consistent with the findings of Malo et al*.* [52], who observed that pro-inflammatory cytokines such as IL-1, IL-6, and TNF-α increase resistin expression in human peripheral blood mononuclear cells.

In the present study, hs-CRP levels were measured both at baseline and after six months of GC supplementation, compared to a placebo. CRP, an acute-phase protein, is released in response to inflammation triggered by IL-6 secretion and is considered an additional marker of metabolic syndrome, with elevated levels often seen in obesity and insulin resistance [43]**.** Our findings indicated that GC supplementation significantly reduced CRP levels. This reduction is likely due to CGA's ability to suppress macrophage infiltration and inhibit the production of inflammatory mediators by down-regulating NF-κB [51, 53]. Moreover, CGA diminished the expression of pro-inflammatory markers like TNF-α and IL-6, ROS and RNS in type 1  and 2 diabetes in several animal and human studies.

The present study found that supplementation with GC significantly reduced TNF-α levels compared to the placebo group. Additionally, TNF-α levels were positively correlated with other inflammatory biomarkers. These findings are consistent with the work of Tzanavari et al*.* [54], who reported that obesity leads to macrophage infiltration in adipose tissue and increased production of the pro-inflammatory cytokine TNF-α, which correlates with adiposity and insulin resistance. Targeting TNF-α and/or its receptors may therefore be a promising approach for treating type II diabetes and insulin resistance [51, 54]. Furthermore, CGA has been shown to exert cardioprotective effects by inhibiting the Nrf2/HO-1 and TGF-β/Smads signaling pathways [53]**.**

GC has the ability to scavenge free radicals and enhance antioxidant capacity both in vivo and in vitro. This effect was supported by our study, which found that serum levels of MDA, a marker of lipid peroxidation, were significantly lower after six months of GC supplementation (800 mg daily) compared to the placebo. The antioxidant properties of CGA are primarily mediated through the transcription factor nuclear factor-E2-related factor 2 (Nrf2), which regulates phase II detoxifying enzymes, including superoxide dismutase, glutathione peroxidase, and glutathione reductase [51, 53, 55]. The antioxidant activity of GC plays a crucial role in preventing the oxidation of LDL-C, particularly in patients with dyslipidemia [55, 56].

In the present study, serum adiponectin level was increased by GC supplementation compared with placebo. These results were consistent with Lukitasari et al. [57], who reported that CGA can induce the transcriptional activity of PPAR-γ and consequently adiponectin production by adipose tissue. Moreover, chlorogenic acid is a potential agonist of PPAR-γ regulating glucose homeostasis and increasing insulin sensitivity of peripheral tissues thus preventing type II diabetes [58]**.**

This study is the first to investigate the impact of green coffee bean extract supplementation on miR-133a and miR-155. Recently, miRNAs have been identified as key biological regulators with the potential to influence inflammation through various pathways [59]**.** Our findings revealed a significant reduction in miR-155 levels following six months of green coffee treatment compared to the placebo group. Additionally, miR-155 showed a significant correlation with fasting blood glucose (FBG), HbA1c, HOMA-IR, total sialic acid, and miR-133a. Prior to the intervention, miR-155 was up-regulated in obese patients. These results align with previous studies indicating that miR-155 contributes to adipose tissue dysfunction and insulin resistance [60, 61]**.** Furthermore, the study found that elevated miR-155 levels were associated with increased resistin, hs-CRP, TNF-α, and total sialic acid. These observations are consistent with earlier research showing that resistin levels are notably higher in the plasma of miR-155-/-/ApoE-/- obese mouse models compared to ApoE-/- mice, indicating a miR-155-mediated suppression of inflammation in adipose tissue [62].

Ohishi et al. [63] discovered that CGA may block CD36 via AMPK activation, resulting in decreased lipid absorption and transport. CGA also raised miR-122 levels, a liver-specific miRNA that is crucial for liver homeostasis. This indicated that CGA may suppress lipogenesis and fatty acid synthase via post-transcriptional mechanisms [64].

We also assessed miR-133a expression in obese patients with metabolic syndrome (MetS) before and after treatment with green coffee (GC). Initially, miR-133a was up-regulated in these patients, but GC supplementation for six months significantly reduced its levels. Furthermore, serum levels of miR-133a were positively correlated with fasting blood glucose (FBG) and HbA1c. These findings are consistent with previous research [63, 65, 66], which has identified circulating miR-133a as a biomarker for myocardial damage. Elevated serum levels of miR-133a have been observed in patients with severe myocardial injury and adverse cardiovascular events [67]. Additionally, Kim et al*.* [68] demonstrated that CGA protects against alcohol-induced liver injury in mice by modulating hepatic miRNAs that regulate mitochondrial redox systems.

## Conclusion

This study revealed the beneficial effects of green coffee bean extract administration on the improvement of BMI, HOMA-IR index, lipid profile, fasting blood glucose, and modulation of inflammatory biomarkers and miRNAs related to obesity. The present study was the first study that showed the effect of green coffee been extract supplementation on miR-133a and miR-155 expression. Also, the antioxidant effect of chlorogenic acid, the main constituent of GC suppressed the oxidative stress and inflammation. In addition, the inflammatory biomarkers, miR-133a and miR-155 together may have diagnostic or predictive value, which will be of clinical importance in obese patient`s follow-up.

### Limitations

Nevertheless, the present study has limitations. First, the relatively small sample size for analysis, which needs further investigation in more cases in multi-centers setting. Second, longer duration of treatment may be warranted.

## Data Availability

The data that support the findings of this study are available on request from corresponding author.
